# Flight efficiency is a key to diverse wing morphologies in small insects

**DOI:** 10.1098/rsif.2021.0518

**Published:** 2021-10-20

**Authors:** Thomas Engels, Dmitry Kolomenskiy, Fritz-Olaf Lehmann

**Affiliations:** ^1^ Department of Animal Physiology, Institute of Biosciences, University of Rostock, Albert-Einstein-Str. 3, 18059 Rostock, Germany; ^2^ Center for Design, Manufacturing and Materials, Skolkovo Institute of Science and Technology, 30 Bolshoi Boulevard, Moscow 121205, Russia

**Keywords:** insect flight, bristled wings, unsteady aerodynamics, Rankine–Froude efficiency, numerical simulation

## Abstract

Insect wings are hybrid structures that are typically composed of veins and solid membranes. In some of the smallest flying insects, however, the wing membrane is replaced by hair-like bristles attached to a solid root. Bristles and membranous wing surfaces coexist in small but not in large insect species. There is no satisfying explanation for this finding as aerodynamic force production is always smaller in bristled than solid wings. This computational study suggests that the diversity of wing structure in small insects results from aerodynamic efficiency rather than from the requirements to produce elevated forces for flight. The tested wings vary from fully membranous to sparsely bristled and were flapped around a wing root with lift- and drag-based wing kinematic patterns and at different Reynolds numbers (*Re*). The results show that the decrease in aerodynamic efficiency with decreasing surface solidity is significantly smaller at *Re* = 4 than *Re* = 57. A replacement of wing membrane by bristles thus causes less change in energetic costs for flight in small compared to large insects. As a consequence, small insects may fly with bristled and solid wing surfaces at similar efficacy, while larger insects must use membranous wings for an efficient production of flight forces. The above findings are significant for the biological fitness and dispersal of insects that fly at elevated energy expenditures.

## 1. Introduction

In the course of evolution, many insect species have gone through a process of extreme reduction in body size. Within a single order, insects often cover an approximately 100-fold range of different sizes, resulting in body lengths ranging from several centimetres to fractions of a millimetre. The process of miniaturization is a result of ancestral genetic traits such as modification in the activity of the moulting hormone [1] as well as traits derived by environmental factors such as nutrition, temperature and oxygen level [2–4]. These factors lead to traits such as structural reduction, increased variability and in many cases morphological novelty. Although many small insect species rely on flapping flight for locomotion, they suffer from their small size in different ways. This includes a tremendous reduction of neurons for vision and flight control, a decrease in flight muscle efficiency due to a decrease in muscle fibre length and elevated energetic costs for wing flapping due to elevated viscous friction at low Reynolds numbers (*Re*). Low *Re* below approximately 10 predicts a pronounced decrease in flight performance as well as a decrease in locomotor capacity needed for weight support and manoeuvring flight. Previous work emphasized that the flight of miniature insects results in low lift-to-drag ratios of unity or less. Horridge [5] even suggested that tiny insects have abandoned altogether the aerofoil action and literally swim in air. This is remarkable because flight at the aerodynamic transition from high to low *Re* is challenged by pronounced costs of overcoming viscous friction on rowing wings. It has even been suggested that some of the smallest insects use their wings only as parachutes aimed to slow down the descent of the animal as it is dispersed by wind [6].

**Figure 1.**
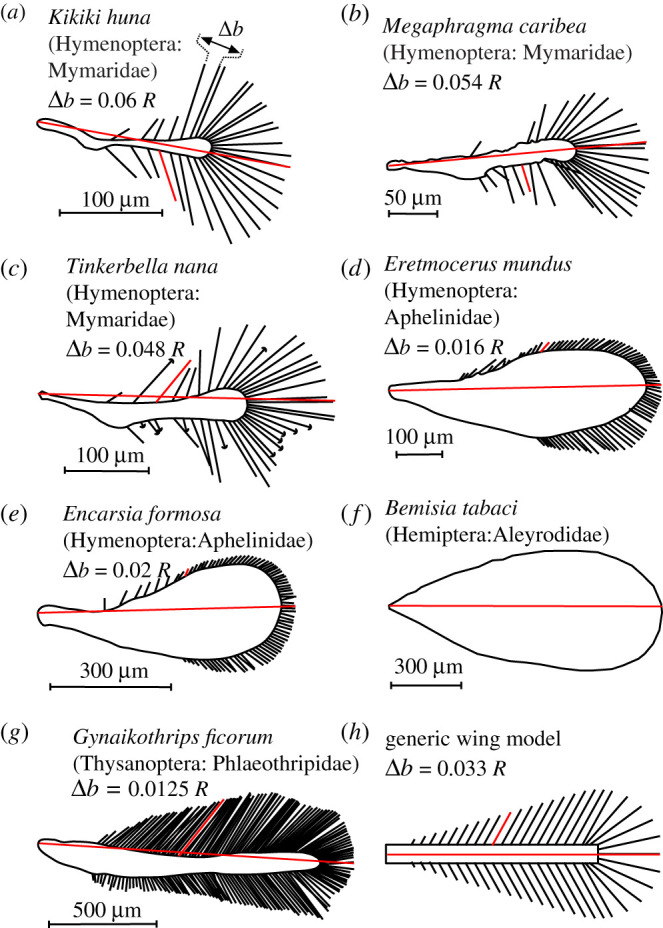
Insect wings and tested model wing. (*a–g*) Wings of small insects, digitized from the literature that is referenced in the electronic supplementary material, table S1. Wings are scaled to the same size and red line indicates the wing's longitudinal axis and wing length *R*. (*h*) Model wing with mean bristle spacing. Bristle spacing Δ*b* was computed as mean distance between two bristle endpoints of bristles in natural wings, excluding the 20% smallest and largest bristles (outliers). The generic wing model covers mean bristle spacing from 0.016*R* to 0.163*R*. Characteristic angle of lateral bristles with respect to the wing's longitudinal axis at the shaft's mid-length is shown for a single bristle (red, §1, electronic supplementary material).

The elevated significance of air viscosity for flight at low *Re* favours certain unique morphological adaptions in small flying insects that are absent in larger species [7]. Ptiloptery is one of these adaptations and refers to wings with long bristles attached to a narrow membranous section (figure 1) [8–10]. Ptiloperty is common but not exclusively observed in small insects. Thus, solid and bristled wings coexist in insects with typical body sizes below 1–2 millimetres, while larger insects only have solid wings surfaces with few exceptions (e.g. *Alucita hexadactyla*). The exact geometry of wing bristles varies tremendously among insect species and little is known on how bristle geometry, solidity ratio and their compliance alter flight performance and energetic costs (figure 1). Ptiloptery is beneficial because the expected reduction of wing mass is thought to save inertial power during wing motion [11]. By contrast, ptiloptery is also adverse, as bristled wings produce less aerodynamic forces than solid wings because air may leak through the wing area [10,12]. In sum, there is no satisfying explanation for the coexistence of solid and bristled wings in small insects.

The majority of large insects have solid wings that consist of thin, impermeable membranes reinforced with veins [13]. The significance of three-dimensional wing shape and outline on lift-generating leading edge vortices (LEVs) has been extensively addressed in the literature [14–18]. In contrast with solid wings, bristled wings feature complex fluid flows that combine flows around each bristle at *Re* below unity with flows around the entire wing at low-to-medium *Re* [10,19]. Analytical studies previously suggested that gap flow between two bristles depends on bristle diameter, spacing and airflow velocity [20]. Bristle spacing has thus a strong impact on the leakiness of wings and their force production [12,21,22]. Wings with an appropriate distance between bristles (gap width) may even act as a solid surface and thus similar to a solid membrane [20]. Experimental work on translating rectangular wings with bristles confirmed this prediction [23]. Although leakiness attenuates force production, bristled wings might be beneficial in gusty exterior flows, reducing peak changes in aerodynamic force production compared to a solid surface [24]. Moreover, bristles may beneficially increase the lift-to-drag ratio during wing flapping [25]. For example, the largest lift-to-drag ratios are reported for wings with an area that consists of 15–30% solid membrane and 70–85% bristles [25]. The latter values are similar to what has been observed in forewings of tiny thrips (figure 1).

Due to experimental limits, early studies described the motion of bristled wings based on theoretical considerations [5,26]. Measured wing kinematics have been published for the parasitoid wasp *Encarsia formosa* [27], small thrips and wasps [6,28], for whiteflies *Bemisia tabaci* during take-off [29], and for several species of the parasitoid wasp *Nasonia*, responding to visual stimuli in a flight simulator [30]. Specific changes in wing kinematics of several small insect species were previously summarized [31]. The wing tip path in small flying insects often follows a figure-of-eight kinematics with pronounced heaving motion and often rely on drag for propulsion [32,33]. The unique wing kinematics of the approximately 1 mm parasitoid wasp *Encarsia formosa* thus appears to be an adaption to elevated viscous friction on wings and body [27]. In the latter species, drag-based rowing motion produces approximately 70% of the vertical force for weight support while the remaining approximately 30% lift is due to the clap-and-fling mechanism [34,35]. Clap-and-fling with bristled wing has been investigated in greater detail [6,12,36], and bristles have been proposed to reduce the forces required to separate the wings during fling motion [37].

Surprisingly, the majority of previous studies on bristled wing aerodynamics have not determined aerodynamic power requirements for wing flapping. Thus, the significance of bristles for aerodynamic efficiency is unsolved, including the question why bristle and solid wings coexist in small but not in larger insects. Our study presents a solution to this enigma using three-dimensional computational fluid dynamics and parametrized wing models with bristle densities typical for small insects. The approach assesses flow fields around individual bristles and the entire wing, aerodynamic forces and moments, aerodynamic power, and Rankine–Froude efficiency of flight. Our conclusion is eventually based on the comparison of efficiencies at different *Re* and thus on the efficiency of how muscle power is translated to vertical force production by morphologically different wings.

## 2. Material and methods

Instead of testing bristled wings of a specific insect, we here used generic wings with bristle spacing similar to those found in various insect species (figure 1*a–g*). Our synthetic wing shapes are motivated by the following considerations: (i) the resemblance with real flapping bristled wings; (ii) a one-parameter family that controls leakiness by the variation of only one geometrical parameter; and (iii) a study focus on aerodynamics and power. Real bristled wings may also vary in relative area of the central membranous part, bristle diameter, secondary outgrowths on the bristles and an asymmetry between the leading and the trailing edges. In addition to aerodynamic performance, these parameters might be optimized with respect to inertia properties and structural stiffness. Our model is a step ahead of previous research on comb wings in rectilinear motion using a more realistic three-dimensional flapping motion. Although the tested wings are simplified and synthetic, our numerical model is sufficient to explore the major effect of flows through bristles and thus overall aerodynamic performance. We measured typical bristle spacing in these species and generated generic bristles at the model wing using a numerical approach for bristle attachment sites and orientation (figure 1*h*; electronic supplementary material, figure S1, §1). We used a snow cone-like outline for the generic wing model because all natural wings have a narrow root with circular wing tips. We created one solid (Δ*b* = 0) and 8-bristled wings with different wing length-normalized bristle spacing (Δ*b*), area coverage and number of bristles (table 1). Bristle diameter was 7.7 × 10^−3^ wing length. The wing model was stiff because bristle bending is likely negligible during wing flapping (§2, electronic supplemental material). Table S1 and §§1 and 3 of the electronic supplementary material summarize morphological and kinematic parameters.

**Table 1.** Geometrical parameters of the tested wings. See also electronic supplementary material, table S1.

bristle spacing Δ*b*	0.163	0.109	0.081	0.065	0.054	0.033	0.022	0.016	0 (solid)
area coverage	0.21	0.23	0.24	0.26	0.28	0.34	0.42	0.51	1.0
number of bristles	9	14	19	24	29	49	74	99	—

The wings in this study are perfectly flat. Earlier work showed that the wing's three-dimensional structure can be separated into large-scale camber-twist and small-scale corrugation [38]. Compared to the expected significance of bristle density on performance, the corrugation component of small wings has little effect on net aerodynamic forces (fruit fly [38]). The central membranous part of bristled wings thus compares to the membranous part of solid wings. Secondary microstructures on bristles may have a substantial impact on bristle drag. However, three-dimensional-printed more realistic bristle models with a cylindrical core and secondary outgrowths generate the same aerodynamic drag as purely cylindrical rods of a larger diameter. Detailed investigations of the significance of bristle diameter on drag are not yet known and a direction for future work.

Owing to the various types of wing kinematics in small insects, we tested two types of wing kinematics at hovering conditions in this study that represent the two extremes of force production in flapping flight: lift-based kinematics with wing flapping in a horizontal stroke plane and figure-of-eight drag-based kinematics, in which vertical lift mainly results from drag on the wing. This approach takes into account that (i) consecutive wingbeats in the same animal may considerably vary [39] and (ii) small insects employ kinematics that are different from the lift-based kinematics observed in larger insects [31]. Although the latter study did not cover insects with bristled wings, it shows that in small insects lift production at the end of each halfstroke typically results from forces parallel to wing motion (drag) and not perpendicular (lift) to it. Another study on the relationship between wing kinematics and *Re* suggests that lift-based kinematics produces larger vertical forces than drag-based kinematics at intermediate *Re*, while vertical forces are similar in lift- and drag-based kinematics at *Re* near zero [33]. Pure lift- and drag-based kinematics can be seen as extreme cases of wing motion because many kinematic patterns in insects combine lift-based and with drag-based force generation [31]. As our main result holds for both kinematic extremes, the use of a specific kinematic pattern that combines lift- and drag-based phases seems to be dispensable for the outcome of this study.

**Figure 2.**
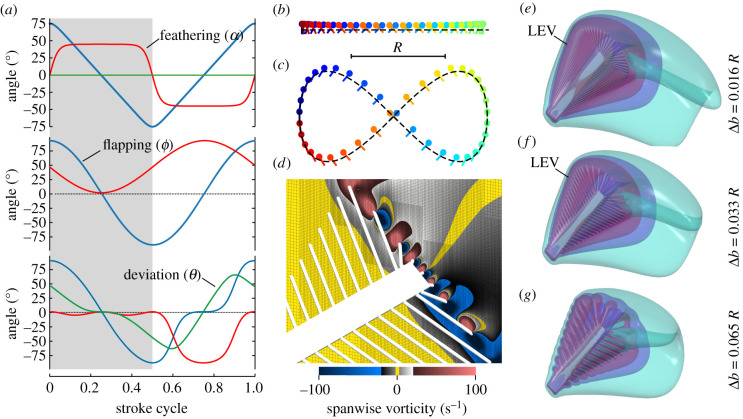
Wingbeat kinematics and flow structures. (*a*) Changes in the wing's feathering angle (red), back-and-forth flapping angle (blue) and vertical heaving angle (green) are shown for lift-based kinematics (upper graph) and drag-based kinematics (middle and lower graph) within a single stroke cycle. Angles are shown for two mathematical notations (middle graph, [40]; lower graph, [41]). (*b,c*) Lollipop diagram of wing chord in *xy*-space during lift-based in (*b*) and drag-based kinematics in (*c*), with dots representing the wing's leading edge. (*d*) Example of two-dimensional spanwise vorticity at bristles, including local grid refinement blocks (thin black lines). (*e,f*) Formation of LEV in bristled wings during lift-based kinematics at 2.9 cycle time (end of translational phase). Colours show semi-transparent iso-surfaces of vorticity magnitude ||*ω*|| = 10*f*, 20*f* and 30*f*. *f*, wingbeat frequency.

**Figure 3.**
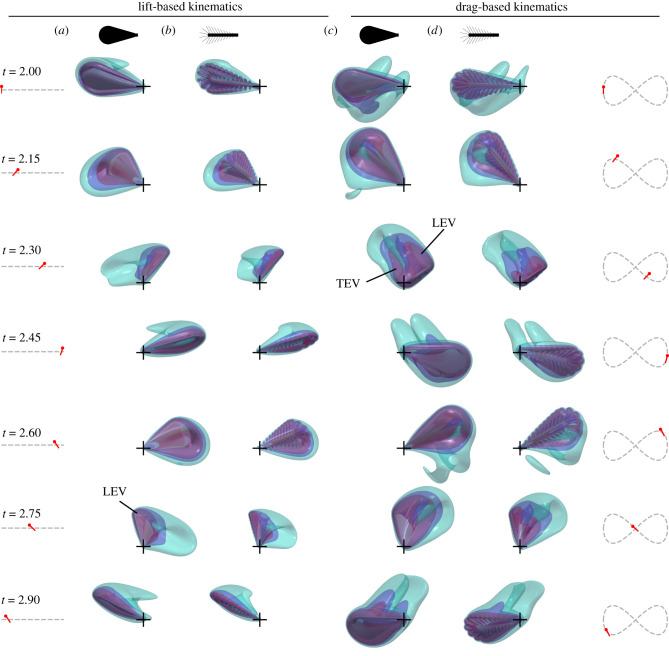
Flow visualization of the third stroke cycle after flight initiation. Flow is generated by a fully membranous wing (*a,c*) and a bristled wing with Δ*b* = 0.065 *(b,d)*, flapped with lift-based (*a,b*, left half) and drag-based (*c,d*, right half) kinematics. Reynolds number is 14. Colours show semi-transparent iso-surfaces of vorticity magnitude ||*ω*|| = 10*f*, 20*f* and 30*f*. *t*, cycle time; *f*, wingbeat frequency. Left and right insets show wing tip trajectories, wing position and the wing's angle of attack.

**Figure 4.**
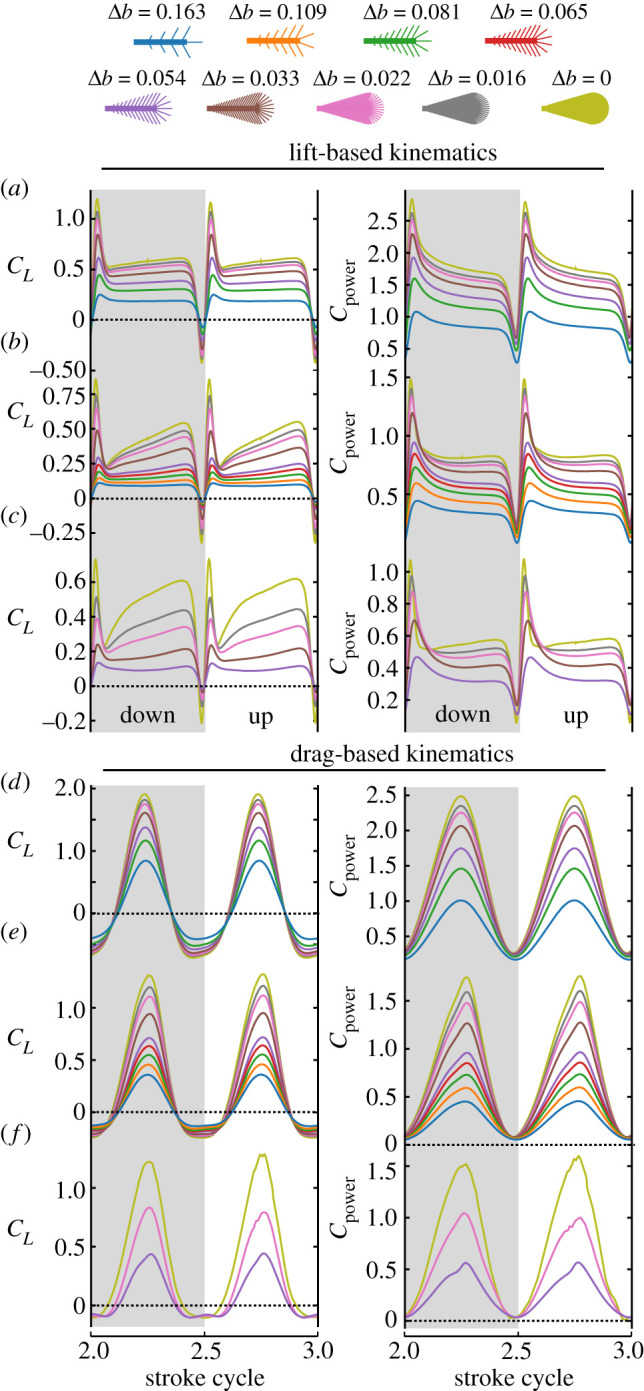
Aerodynamic performance of single model wings. (*a–c*) Performance for lift-based and (*d–f*) drag-based kinematics. Reynolds number is 4 in (*a*), (*d*); 14 in (*b*), (*e*) and 57 in (*c*), (*f*). Grey area indicates the wings' downstroke and colours bristle spacing as shown at the top. Vertical force and aerodynamic power coefficients are plotted for the third stroke cycle.

**Figure 5.**
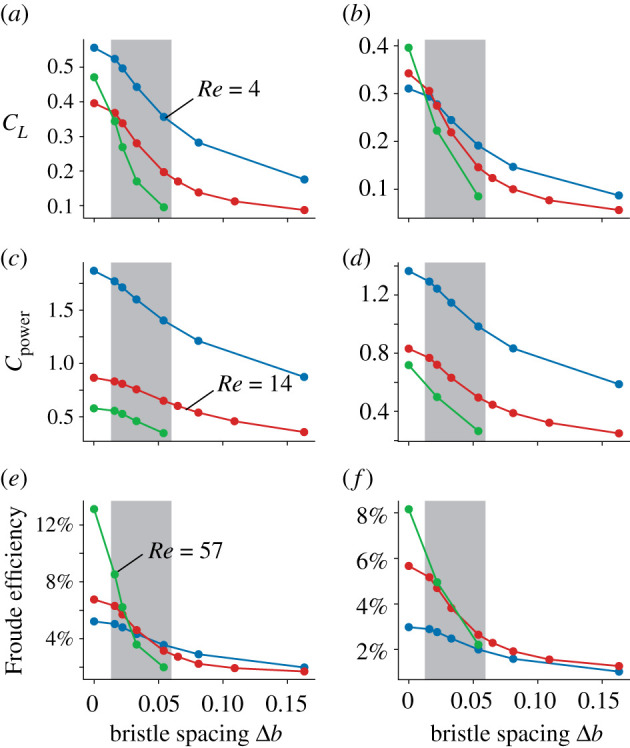
Cycle-averaged performance of a single wing plotted against bristle spacing. Mean (*a,b*) vertical force coefficient, (*c,d*) aerodynamic power coefficient and (*e,f*) Rankine–Froude efficiency of wings flapping at three Reynolds numbers (*Re*). Data stem from the third stroke cycle. Performance for lift-based kinematics is shown in (*a*,*c*,*e*), and for drag-based kinematics in (*b*,*d*,*f*). The highlighted region corresponds to the range of bristle spacing of the real wings from figure 1.

Figure 2*a*–*c* shows both kinematics patterns, and details on kinematics and the derivation of *Re* are presented in §§3–5 of the electronic supplementary material. Back-and-forth flapping amplitude was 150° with symmetrical wing rotation at the stroke reversals and wing feathering angle was 45° and 90° at a mid-half stroke for lift- and drag-based kinematics, respectively. In this study, we defined the *Re* as *Re* = *R*^2^*f*/*ν_air_* (§5, electronic supplementary material). *Re* for wing flapping in the natural archetypes (electronic supplementary material, table S1) varies between approximately 7.0 and approximately 24.0 and *Re* for simulations was thus 4, 14 and 57. Notably, wingbeat frequency varies among individuals as well as within one specimen depending on the flight condition. Thus, *Re* shown in electronic supplementary material, table S1, are approximations and we always selected the lowest *Re* for our simulations. Medium and high *Re* were obtained from a fourfold increase of the smallest numbers. The data in figures 3–5 stem from the simulation of both kinematic patterns in a single wing of the model insect. As mentioned in the Introduction section, however, bristled wings potentially reduce the forces to pull wings apart during clap-and-fling kinematics (§4, electronic supplementary material). Changes in flight efficiency with changing bristle density might thus result from the interaction of left and right wing and not from the changing performance of a single wing. To consider this idea, we flapped also two wings using lift-based kinematics and compared the effect of wing–wing interaction in various bristled wing with forces and power determined in a single wing.

All data are calculated from a computational fluid dynamic approach using our freely available, open-source, in-house code WABBIT (Wavelet Adaptive Block-Based solver for Insects in Turbulence) [42]. The code was originally designed to handle multiscale turbulence in insect flight but it can also be used for multiscale geometries as they occur in bristled wings. Here, we use the code with explicit second-order finite differences to solve the artificial compressibility equations [43]. A detailed description of how the code has been adapted to bristled wing simulations at low *Re* is presented in §6 of the electronic supplementary material. This material also includes a validation of both the numerical method and the independence of the numerical grid size in bristled wing simulations. We performed a total of 40 simulation runs using up to nine wings at three *Re*. In all simulations, we computed three consecutive wing beat cycles because additional control simulations indicated that cycle-averaged forces and power change only little after the initial wingbeat cycle. Vertical force and aerodynamic power are presented as dimensionless coefficients:

2.1 $$C_{L}=\frac{F_{z}}{0.5\rho u_{tip}^{2}A_{w}}$$

and

2.2 $$C_{\mathrm{power}}={\frac{P_{aero}}{0.5\rho u_{tip}^{3}A_{w}}}_{},$$

with *A_w_* = 0.289*R*^2^ the surface of the solid wing, *ρ* the density of air, *R* the wing length, *f* the wingbeat frequency and *u_tip_* the mean wingtip velocity, i.e. 5.2*Rf* and 7.5*Rf* in lift- and drag-based kinematics, respectively.

## 3. Results and discussion

Figure 3 shows the time evolution of vorticity around a solid wing at *Re* = 14 for lift- (figure 3*a*) and drag-based (figure 3*c*) kinematics. The planar motion in lift-based kinematics induces the formation of a stable pronounced LEV during the translation phase of wing motion. At the stroke reversals, the vortex is not shed into the wake but quickly dissipates at the wing owing to elevated viscosity at low *Re*. As a consequence, vortices remain localized near the wing that reduces the minimum distance for wake interference between left and right wing. In contrast with lift-based kinematics, flow topology of drag-based kinematics shows fundamental changes. Due to the increase in mean wing velocity owing to the longer figure-of-eight wing path, the vortical flows extend to a larger fluid volume compared to the lift-based wake. During the translation phase, the wing moves downwards at a high angle of attack (2.15 and 2.30 stroke cycle, figure 3*c*) and simultaneously generates LEV and trailing edge vortices (TEV). For the same kinematics and *Re*, figure 3*b,d* shows the vorticity generated by a bristled wing with Δ*b* = 0.065. The qualitative flow field is similar but fluid is leaking through the bristled wing. This results in a more confined wake and less vorticity is generated at the leading edge (lift- and drag-based) and trailing edge (drag-based kinematics). The vortex cores are significantly less developed. Bristled wings produce vorticity at each single bristle (figure 2*d*) that may fuse into a wing-wide vorticity field. In wings with small bristle distance (Δ*b* = 0.016, figure 2*e*; Δ*b* = 0.033, figure 2*f*), vortices at leading edge bristles form a coherent LEV. This process is attenuated at larger bristle spacing at which a coherent LEV is broadly absent (Δ*b* = 0.065, figures 2*g* and 3*b,d*). Bristle spacing thus alters the fine structure of vortex development at both leading and trailing wing edges. This change in vortex topology leads to changes in lift production and power requirements for flight.

Figure 4 summarizes our result on vertical force production and aerodynamic power requirements for wing flapping. The figure shows the time evolution of both coefficients in all tested wings. Wings flapped with lift-based kinematics exhibit large force peaks during the stroke reversals due to rotational circulation (figure 4*a*–*c*). Forces during the translational part of wing motion are always smaller than during wing rotation. With increasing *Re*, vertical force production increasingly collapses after wing rotation followed by an increasing recovery during the translational phase of wing flapping. Throughout the flapping cycle, the vertical force coefficient decreases with increasing bristle spacing. Thus, as predicted from aerodynamic theory, solid wings produce largest vertical forces at largest power for all tested *Re* (4, figure 4*a*,*d*; 14, figure 4*b*,*e*; 57, figure 4*c*,*f*).

Time evolution of force coefficient during drag-based kinematics tremendously differs from lift-based kinematics and peaks for all tested wings and *Re* at mid-half stroke (figure 4*d*–*f*). In absolute numbers, the corresponding peak force *F_z_* is approximately 14 times higher than the corresponding value during lift-based kinematics. The peak coincides with the phase at which wing chord is normal to the direction of wing motion and drag is thus maximum. Notably, at the stroke reversal force is negative because of the wing's zero angle of attack and an unfavourable direction of induced flow. Both factors attenuate mean vertical force coefficient and thus aerodynamic efficiency. With increasing *Re*, negative force decreases in magnitude due the decrease in skin friction (figure 4*d*–*f*). Similar to lift-based kinematics, vertical forces strictly decrease with increasing bristle distance. To assess the cost of force production, we also calculated aerodynamic power coefficient for wing flapping. We found that the temporal development in power requirements is broadly similar to the structure of vertical force production with maximum power requirements at the stroke reversals (right column, figure 4).

Locomotor systems in animals are typically shaped by the evolutionary pressure to lower the costs of propulsion [44]. The relative costs of weight-supporting lift production in flapping flight of insects depends on the efficiency with which an animal converts flight muscle power into aerodynamic lift. Rankine–Froude efficiency (figure-of-merit, *η*) characterizes this performance in flight systems (see §7, electronic supplementary material) [45]. Figure 5 shows that vertical force, aerodynamic power and efficiency decrease with increasing bristle spacing. A solid wing (Δ*b* = 0) that flaps with lift-based kinematics is most efficient (*η* = 13.7%) at *Re* = 57 and least efficient (*η* = 5.2%) at *Re* = 4 owing to viscous drag (figure 5*e*). Viscous drag even more attenuates efficiency in drag-based kinematics (*η* = 8.2%, *Re* = 57; *η* = 3.0%, at *Re* = 4). As expected, these efficiencies are relatively low compared to insect wings flapping at larger *Re*. For example, efficiency in the blowfly *Calliphora vomitoria* (Re∼1000) amounts to approximately 23% [38]. From this point of view, insects that fly at large (*Re* = 57) and very low *Re* (*Re* = 4) should employ lift-based kinematics to minimize their energetic expenditures. In insects with medium *Re* (*Re* = 14), by contrast, flight efficiency is similar for drag- and lift-based kinematics (*η* = 6.8% versus *η* = 5.7%; figure 5*e*,*f*). Notably, owing to geometric and kinematic simplification, the model wings tested in this study may produce less lift than similarly sized real insect wings. For example, weight-supporting lift coefficient of *Paratuposa placentis* [46] is 1.5 and 0.7 during lift- and drag-based flight, respectively (cf. equations (2.1)–(2.2)). The latter values are not attained by the data shown in figure 5.

The decrease in rate of performance with increasing bristle spacing is most striking in figure 5 and leads to a possible explanation why bristled and solid wings coexist in small but not in larger insects. Most importantly, this conclusion is independent of the kinematic pattern. In general, as drag-based kinematics distributes force production less evenly throughout the stroke, efficiencies are smaller in drag- than in lift-based kinematics (e.g. solid wings, 14% versus 8%; figure 5*e*,*f*). This finding confirms previous results of two-dimensional simulations on the disadvantages of drag-based flight at elevated *Re* [33]. In both kinematics, we found that Froude efficiency rapidly decreases with increasing bristle spacing at all *Re* (slopes; lift-based kinematics, 0.31, 0.71, 2.07; drag-based kinematics, 0.19, 0.60, 1.09; *Re* = 4, *Re* = 14, *Re* = 57, respectively; mean *R*^2^ = 0.94). This decrease is 5.6–6.6 times larger in large insects (*Re* = 57) than in small insects (*Re* = 4). Thus, at a spacing of Δ*b* = 0.033 (figure 5*e*), a bristled wing is more efficient at *Re* = 4 and *Re* = 14 than at *Re* = 57 despite its higher energetic costs to overcome viscosity. In other words: an area change from solid (Δ*b* = 0) to bristled (Δ*b* = 0.033) results in approximately 56% loss in efficiency in large insects (*Re* = 57) but only in an approximately 32% (*Re* = 14) and approximately 17% loss (*Re* = 4) in small insects. The steep loss in performance of bristled wings reflects a strong evolutionary pressure on large insects to evolve solid wing surfaces for flight. The shallow slope in efficiency of small insects (*Re* = 4 and 14), by contrast, lowers this pressure and allows a larger variety of wing surface designs.

As mentioned above, it has been suggested that bristles might be an adaption to clap-and-fling kinematics because bristles lower the forces needed to pull the wing surfaces apart during fling motion (Hymenoptera: Aphelinidae [34] and Mymaridae [37]; Thysanoptera: Phlaeothripidae [25]; Diptera: Drosophilidae [47]). We thus repeated the simulations on two wings with Δ*b* = 0, 0.033 and 0.081 at *Re* = 14, using a modified lift-based wingbeat kinematics (flapping angle amplitude = 180°) with near-clap conditions (§4, electronic supplementary material). We found that the evolution of vertical force and aerodynamic power (electronic supplementary material, figure S3) is quite similar to the data in figure 4. However, instantaneous peak force *F_z_* during stroke reversal is approximately 3.3 and aerodynamic power *P*_aero_ is approximately 4.5 times higher compared to a single wing. Force enhancement decreases with decreasing bristle spacing and disappears when bristle spacing exceeds approximately Δ*b* = 0.081. The latter finding is consistent with previous research [37]. Cycle-averaged vertical force, mean power and Rankine–Froude efficiency decrease with increasing bristle spacing (total force coefficient, 1.47, 0.83, 0.37; total power coefficient, 3.6, 2.6, 1.7; efficiency, 10.5%, 6.1%, 2.9%; Δ*b* = 0, Δ*b* = 0.033, Δ*b* = 0.081, respectively) that is similar to what we find in a single wing. However, the rate of change in efficiency with changing bristle spacing is approximately 23% higher for clap-and-fling kinematics compared to a single flapping wing (−0.92 versus −0.71; figure 5*e*). This potentially means that insects with fully membranous wings and using clap-and-fling kinematics face a trade-off, i.e. elevated flight efficiency at the cost of elevated peak power requirements during the stroke reversals.

## 4. Conclusion

In conclusion, although numerous studies focused on the secrets of flight in miniature insects, most experimental data that elucidated wing motion and aerodynamics were obtained from larger insects. Previous two-dimensional numerical studies on the aerodynamics of bristle wings showed that the flow past evenly spaced cylinder lattices reduces aerodynamic force production in bristled wings [21,22]. This effect is broadly due to the additional costs to overcome viscous drag during wing flapping at low *Re*. Our data offer an explanation for the coexistence of bristled and solid wings in insects, assuming that aerodynamic efficiency rather than maximum force production is key to flight in miniature animals. Nevertheless, other studies suggested explanations based on the energy consumption to develop solid wing surfaces during the animal's development. The latter idea results from the assumption that wings evolved from gills [48]. Gills are functionally leaky organs to extract oxygen from water also used for locomotion, protection and chemoreception. While small insects might have kept the comb-like structure from their early relatives, wings evolved into membranous surfaces when animals developed larger body sizes [44]. Our data support the assumption that efficiency might have shaped the evolution of wing design suggesting membranous wings in insects larger than several millimetres, while wing design in miniature insects is shaped by aerodynamics and factors other than flight efficiency.

## Data accessibility

Data are available within the supplementary material file.

The data are provided in the electronic supplementary material [49].

All data needed to evaluate the conclusions in the present paper and the electronic supplementary material are available on request from the corresponding author.
